# The effect of individuals' oral hygiene habits and knowledge levels on peri-implant health and disease: a questionnaire-based observational study

**DOI:** 10.1186/s12903-024-04211-y

**Published:** 2024-04-11

**Authors:** Tuğba Şahin

**Affiliations:** Division of Periodontology, Faculty of Dentistry, Abant İzzet Baysal University, Bolu, Turkey

**Keywords:** Awareness, Oral health, Oral hygiene, Peri-implantitis, Surveys and questionnaires

## Abstract

**Background:**

Peri-implant disease and health are associated with microbial dental plaque. Therefore, oral hygiene plays a role in preventing and treating these diseases. This study aimed to determine the relationships among knowledge of peri-implant status, oral hygiene habits, and peri-implant disease and health.

**Methods:**

A total of 144 implants in nonsmokers with controlled systemic disease were included in the study. Peri-implant disease and the conditions of the implants were determined with periodontal indices and radiographs based on the 2017 World Workshop on the Classification of Periodontal and Peri-implant Diseases and Conditions and The EFP S3 level clinical practice guideline. Individuals were asked 66 questions regarding demographic information, oral hygiene habits and history, and knowledge of peri-implant diseases. One-way ANOVA was used to compare the three peri-implant disease and condition categories.

**Results:**

There was a significant difference between groups regarding toothpaste type (*p* < 0.05). Gum protection toothpaste was greater in the peri-implant health group. Patients' use of interdental products was very low; often, no products were used for implant prosthesis. There was no significant difference among the groups regarding oral hygiene product use or oral hygiene habits (*p* > 0.05). There was a significant difference between groups regarding frequency of visit (*p* < 0.05). The frequency of visits to the dentist for pain was greater for individuals with peri-implantitis. There is a significant difference between the groups' answers for the causative and initiating factors of peri-implant disease (*p* < 0.05). The peri-implant health group answered that microbial dental plaque is the most crucial initiating factor of peri-implant diseases, and bleeding on probing is the most critical determinant of peri-implant diseases at a higher rate than the other groups.

**Conclusions:**

Patients' oral hygiene habits and knowledge levels are almost similar according to peri-implant status. Knowledge does not reflect a patient's oral hygiene behavior. Clinicians should ensure that individuals' oral hygiene practices align with their increased awareness regarding peri-implant illnesses.

## Introduction

Dental implants are a well-recognized method of replacing lost teeth [1]. Peri-implant diseases and conditions are divided into four categories according to the Tonetti classification: peri-implant health, peri-implant mucositis, peri-implantitis, and soft-hard tissue deficiencies [2]. Peri-implant health does not include erythema, bleeding on probing, swelling, or suppuration, and peri-implant health is synonymous with implant success [3, 4]. Inflammation of the mucosa around endosseous tissue without bone loss is known as peri-implant mucositis, which is a pathological disease [5, 6]. Peri-implantitis is a pathological disorder characterized by inflammation of the periodontal tissues, resulting in the gradual loss of implants and supporting bone [7]. The relationship between microbial dental plaque and gingivitis or periodontitis has been indicated by “classic” experimental gingivitis/periodontitis studies. This situation is thought to be the same for implants [4]. The peri-implant microbiota represents a qualitatively inferior but quantitatively superior bacterial ecosystem for some bacterial genera compared to the periodontal microbiota, showing that a progression from a healthy state to peri-implantitis causes changes in microbiota composition in the absence of specific disease-causing bacteria [8]. The recommended approach for treating peri-implant mucositis is a combination of effective patient-administered biofilm control [9] and professional mechanical biofilm removal [10]. Ozone, glycine/erythritol, probiotics, and chlorhexidine can be used in addition to mechanical debridement in non-surgical peri-implant treatment [11]. Peri-implantitis occurs due to the accumulation of plaque caused by inadequate dental hygiene. Renvert et al. (2015) reported that more than 50% of patients with implants affected by peri-implantitis lacked the ability or opportunity for adequate oral hygiene [12]. The majority of implant issues arise due to challenges in maintaining oral hygiene [13]. Hence, oral hygiene practices effectively eliminate microbial dental plaque [14]. Following the right maintenance protocol may assist implant patients in maintaining good oral hygiene and reduce the chance of subsequent implant failure from both a clinical and sociopsychological attitude-related perspective [15].

Peri-implantitis arises from the accumulation of plaque caused by inadequate dental hygiene [16]. In most cases, information about how to care for prosthesis or the hazards of not maintaining basic hygiene surrounding the implants needs to be provided. A lack of information provided by dentists has been linked to unhealthy peri-implant diseases [17]. Optimizing dental implant therapy procedures and increasing oral hygiene awareness among the general public are crucial [18]. Providing information on proper oral hygiene techniques and reinforcing knowledge and adherence to these practices results in a reduction in dental plaque levels [19].

Oral health knowledge is considered to be crucial for developing healthy behaviors, and it has been shown that there is an association between increased knowledge and better oral health [20]. Previous surveys of patients’ implant experiences have investigated patient perceptions [21, 22] experiences [23], and awareness of dental implant treatment [24]. The information regarding implants mostly relates to the nature of the treatment [25]. Research on the assessment of peri-implant conditions via interdental cleaning is accessible [17]. Previous studies have assessed the oral hygiene practices and knowledge of individuals with dental implants using a limited number of survey questions. The measurements do not correspond to to the conditions around the implant or the individuals receiving the implant.

When individuals with peri-implant disease and periodontal disease are compared, some similarities are observed. Therefore, the aim of this study was to determine the relationships among oral hygiene habits and knowledge levels, peri-implant disease status, and the conditions of patients with implants.

## Materials and methods

### Study settings

The Clinical Research Ethics Committee of the Bolu Abant İzzet Baysal University, Bolu approved this study (2022/162). Informed consent was obtained from all participants before enrollment in the study. Prior to participation, all patients were informed about the purpose and the questionnaire, and written informed consent was obtained in accordance with the Helsinki Declaration [26]. Details on the adherence to the STROBE criteria for cross-sectional studies are reported.

The study was completed at the Faculty of Dentistry's Periodontology Department between July 2022 and November 2023 and included individuals receiving dental check-ups or treatment.

### Sample size calculation

According to the power analysis results of the G Power program (G * Power 3.1 software; Heinrich Heine University, Düsseldorf, Germany) performed within the scope of the study; for the chi-square analysis to be performed for the comparison of multiple responses given by the 3 groups, it was determined that a minimum of 108 samples were required at the level of 0.40 effect (w), 0.90 power (1-β) with a margin of error (α) of 0.05 at 3 degrees of freedom.

### Study design, inclusion and exclusion criteria

The medical history of patients with implants who visited the clinic was recorded, and their overall health was assessed. Individuals with uncontrolled systemic diseases, diabetes, or bruxism, individuals who smoked, and individuals who were breastfeeding or pregnant were excluded. Patients with mental disorders that could make it difficult to complete the questionnaire were excluded from the study. A history of nonsurgical or surgical periodontal therapy in the last six months and health and disease status were determined by taking indices from individuals with implants with fixed implant-supported prosthesis for whom it had been at least one year since functional prosthetic loading. Diagnoses were based on the 2017 World Workshop on Classifying Periodontal and Peri-Implant Diseases and Conditions and the 2023 EFP Guideline [27, 28]. The patients were divided into three groups according to following conditions: peri-implantitis, peri-implant mucositis, and peri-implant health. A total of 144 implants were included in the study, with 48 implants in each group.

### Peri-implant disease measure

The clinical oral examination was carried out by one calibrated examiner (T.Ş.) and included the assessment of implants. The implant-related examination included the following measurements: plaque index [29], probing pocket depth, gingival index [30], bleeding on probing [31], clinical attachment level, keratinized tissue thickness, and keratinized tissue width. The peri-implant probing measurements were carried out using a manual UNC-15 periodontal probe (PCP15; Hu-Friedy, Chicago, IL, USA) at 6 sites/implant. Plaque and gingival indices were calculated by giving assigning a value ​​from 0 to 3. The presence or absence of bleeding was measured for each implant after probing. The probing depth, measured at six sites per implant, was defined as the distance between the base of the sulcus and the margin of the gingiva.

### Questionnaire

Participants who did not answer all of the survey questions used in the research or who did not answer the survey questions following the specified instructions were excluded from the study. The data were collected by using self-administered structured questionnaires, some of which were adapted from related literature [32, 33]. The answer options for the questions ranged from two to eighteen. There was also "do not know" option for questions containing information.

A questionnaire was used to gain insight into the knowledge, attitudes, and behaviors related to dental care habits among people with implants in three groups in Turkey. Individuals who had implants were asked to answer a total of 66 multiple-choice questions, including 11 related to general and demographic information (such as age, sex, and sex), 36 related to oral hygiene habits (attitudes toward toothbrush care, etc.), and 19 related to awareness/knowledge of toothbrush care and maintenance.

In the first section data on demographic characteristics were collected age, sex, marital status, educational status of the family, whether the individuals were health workers, and with whom the individuals were assessed. The second section, which examined oral habits included were examined, many questions were asked, such as where the individuals learned to brush, systemic disease, smoking, tooth brushing frequency, duration, products, how they brush, and dentist appointments. In the third part, where the participants’ the knowledge was measured, questions such as the factor that initiated periodontal disease, its symptoms and the effect of systemic disease, how many minutes, how often, and how they brushed their teeth were asked.

A clinician (T.Ş.) was administered the survey to the patients. All the questionnaires were provided in printed formats, and the primary language was Turkish.

### Statistical method

The research analysis was conducted by using the SPSS 26 (SPSS Inc, Chiago, IL, USA) statistical program. In the present study, a total of 48 implants in each of the three groups. A total of 144 implants were analyzed. In the comparison of the study groups according to the data, one-way ANOVA test used since it meets the parametric test criteria for continuous measured data. Chi-square analyse were used to analyze the differences among the groups for the answers provided for the survey questions. *p* < 0.05 was considered to indicate statistical significance.

## Results

### Demographic characteristics

The mean age of the individuals was 46 ± 17.9 years; 44.8% were women, and 55,2% were men. There was no statistically significant difference among the groups regarding the body mass index value (BMI), sex, gender, marital status, income level, parental status, academic status of the participants’ parents, health worker status, or the person with whom participants’ lived (*p* > 0.05). There was a statistically significant difference among the groups regarding education level.

### Oral hygiene training and dentist history

The patients had previously been able to receive information and training on tooth brushing, interdental cleaning, and general oral care from a dentist and had obtained information and training on tooth brushing, interdental cleaning, and available oral care from anywhere other than a dental office. There was no statistically significant difference among the groups in terms of being shown how to brush on a dental model or their teeth by a dentist, the use of the disclosing tablet by a dentist, or the status of taking records/measurements to determine periodontal disease/oral hygiene habits/implant status (*p* > 0.05). In all the groups, most participants stated that they had visited a dentist before and received education from a dentist.

### Health status of ındividuals and habits affecting health

There was no statistically significant difference among the groups regarding prior psychiatric diseases, systemic disorders, or current systemic disorders (*p* > 0.05).

There was a statistically significant difference among the groups regarding smoking status and the quantity of cigarettes consumed (*p* < 0.05).

### Tooth brushing habits and products of participants

There was no statistically significant difference among the groups regarding tooth brushing frequency, duration, products used, or technique (*p* > 0.05) (Table 1).

**Table 1:** Comparison of participants’ tooth brushing habits and products by group

		Groups			Total	*p*
		Peri-implantitis	Peri-implant mucositis	Peri-implant health		
How often do you brush your teeth?	Once a month	1	0	0	1	0.699
		4.00%	0.00%	0.00%	1.70%	
	2–3 times each month	0	2	1	3	
		0.00%	10.00%	7.70%	5.20%	
	2–3 times a week	2	2	1	5	
		8.00%	10.00%	7.70%	8.60%	
	1 time per day	11	6	3	20	
		44.00%	30.00%	23.10%	34.50%	
	2 or more times per day	11	10	8	29	
		44.00%	50.00%	61.50%	50.00%	
What is your tooth brushing time?	less than 1 min	6	3	1	10	0.650
		24.00%	15.00%	7.70%	17.20%	
	between 1–2 min	13	9	8	30	
		52.00%	45.00%	61.50%	51.70%	
	more than 2 min	6	8	4	18	
		24.00%	40.00%	30.80%	31.00%	
Which one(s) of the following products do you use (You can check more than one option)?	Toothbrush	22	19	13	54	0.605
		32.40%	35.80%	41.90%	35.5%	
	Powered toothbrush	3	2	1	6	
		4.40%	3.80%	3.20%	3.9%	
	Toothpaste	17	14	7	38	
		25.00%	26.40%	22.60%	25.0%	
	Dental floss	7	5	5	17	
		10.30%	9.40%	16.10%	11.2%	
	İnterdental brush	6	5	3	14	
		8.80%	9.40%	9.70%	9.2%	
	Mouthwash	6	2	1	9	
		8.80%	3.80%	3.20%	5.9%	
	Toothpick	7	5	1	13	
		10.30%	9.40%	3.20%	8.6%	
	None	0	1	0	1	
		0.00%	1.90%	0.00%	0.7%	
How do you brush your teeth?	Horizontal (forward–backward)	5	7	2	14	0.133
		20.00%	35.00%	15.40%	24.10%	
	From the tooth-gingiva junction towards the tooth	12	7	6	25	
		48.00%	35.00%	46.20%	43.10%	
	By zig-zag between teeth	3	2	2	7	
		12.00%	10.00%	15.40%	12.10%	
	Random	5	4	3	12	
		20.00%	20.00%	23.10%	20.70%	
Which of the following products do you utilize for your dental prosthesis?	Süperfloss	2	3	4	9	0.207
		8.00%	15.00%	30.80%	15.50%	
	Denture cleansing tablet	0	0	1	1	
		0.00%	0.00%	7.70%	1.70%	
	None	23	17	8	48	
		92.00%	85.00%	61.50%	82.70%	
Which of the following oral hygiene products do you use for your implant?	Oral irrigator	2	0	0	2	0.406
		8.00%	0.00%	0.00%	3.40%	
	Interdental brush	6	7	2	15	
		24.00%	35.00%	15.40%	25.90%	
	Superfloss	2	0	2	4	
		8.00%	0.00%	15.40%	6.90%	
	None	15	13	9	37	
		60.00%	65.00%	69.20%	63.80%	
How often do you use oral hygiene products for your implants?	None	11	10	4	25	0.464
		44.00%	50.00%	30.80%	43.10%	
	Once per month	3	4	1	8	
		12.00%	20.00%	7.70%	13.80%	
	2–3 times a month	2	0	0	2	
		8.00%	0.00%	0.00%	3.40%	
	Once a week	1	1	1	3	
		4.00%	5.00%	7.70%	5.20%	
	2–3 times a week	1	1	3	5	
		4.00%	5.00%	23.10%	8.60%	
	Once a day	4	3	4	11	
		16.00%	15.00%	30.80%	19.00%	
	2 or more times a day	3	1	0	4	
		12.00%	5.00%	0.00%	6.90%	
What is your toothpaste type?	Whitening toothpaste	10	5	2	17	0.029*
		40.00%	25.00%	15.40%	29.30%	
	Anti-caries toothpaste	7	6	3	16	
		28.00%	30.00%	23.10%	27.60%	
	Gum protection toothpaste	4	4	5	13	
		16.00%	20.00%	38.50%	22.40%	
	Anti-sensitivity toothpaste	3	3	3	9	
		12.00%	15.00%	23.10%	15.50%	
	Fluoride toothpaste	1	2	0	3	
		4.00%	10.00%	0.00%	5.20%	
How often do you change your toothbrush?	1–3 months	4	6	4	14	0.563
		16.00%	30.00%	30.80%	24.10%	
	Once every three months	9	7	6	22	
		36.00%	35.00%	46.20%	37.90%	
	3 months-1 year	11	5	3	19	
		44.00%	25.00%	23.10%	32.80%	
	Over 1 year	1	2	0	3	

*p>0.05

There was no statistically significant difference among the groups regarding the product used by the participants for dental prosthesis and implants or the duration of use (*p* > 0.05). Participants mostly did not use any products for their prosthesis or implants (Table 1).

There was a significant difference among the groups regarding the toothpaste type used (*p* < 0.05). While the peri-implantitis group used toothpaste with the highest whitening effect, the peri-implant mucositis and peri-implant health groups used toothpaste that protects the gingiva at the highest rate (Table 1).

### Dentistry visiting habits

There was no statistically significant difference among the groups regarding the last time the participants visited the dentist, the frequency of visiting the dentist for implants, the age at the first dentist visit, or the reasons for visiting the dentist (*p* > 0.05). The majority of people had never seen a dentist. Toothache was the most often selected answer option across all categories (Table 2).

**Table 2:** Comparison of groups in terms of dentist visits

		Groups			Total	*p*
		Peri-implantitis	Peri-implant mukositis	Peri-implant health		
When was the last time you saw a dentist?	0–3 months	6	5	4	15	0.982
		24.0%	25.0%	30.8%	25.9%	
	3–12 months	10	9	5	24	
		40.0%	45.0%	38.5%	41.4%	
	Over 1 year	9	6	4	19	
		36.0%	30.0%	30.8%	32.8%	
How often do you visit the dentist for your implant?	0–3 months	2	1	1	4	0.887
		8.0%	5.0%	7.7%	6.9%	
	3–12 months	5	7	5	17	
		20.0%	35.0%	38.5%	29.3%	
	12–24 months	4	3	2	9	
		16.0%	15.0%	15.4%	15.5%	
	2–6 years	6	2	1	9	
		24.0%	10.0%	7.7%	15.5%	
	None	8	7	4	19	
		32.0%	35.0%	30.8%	32.7%	
What was your first dentist visit age?	0–2 years	0	2	0	2	0.022*
		0.0%	10.0%	0.0%	3.4%	
	2–6 years	4	3	0	7	
		16.0%	15.0%	0.0%	12.1%	
	6–18 years	12	8	5	25	
		48.0%	40.0%	38.5%	43.1%	
	After 18 years	9	7	8	24	
		36.0%	35.0%	61.5%	41.3%	
How often do you visit the dentist?	Once in 6 months	3	7	1	11	0.022*
		12.0%	35.0%	7.7%	19.0%	
	Once in 1 year	9	7	3	19	
		36.0%	35.0%	23.1%	32.8%	
	Once in 2 years	4	6	5	15	
		16.0%	30.0%	38.5%	25.9%	
	Once in 6 years	6	0	2	8	
		24.0%	0.0%	15.4%	13.8%	
	More than 6 years	3	0	2	5	
		12.0%	0.0%	15.4%	8.6%	
For which conditions do you go to the dentist?	Toothache	18	8	7	33	0.373
		72.0%	40.0%	53.8%	56.9%	
	Having prosthesis made or refurbished	1	2	2	5	
		4.0%	10.0%	15.4%	8.6%	
	Scaling	0	3	2	5	
		0.0%	15.0%	15.4%	8.6%	
	Routine dental visit	5	6	2	13	
		20.0%	30.0%	15.4%	22.4%	
	I won't go	1	1	0	2	
		4.0%	5.0%	0.0%	3.4%	

*p>0.05

There was a statistically significant difference in the number of first dental visits and the frequency of dental appointments among the groups (*p* < 0.05). The majority of all groups' initial dental visits occurred when they were six years old or older. The peri-implant health group had longer intervals between visits to the dentist than the peri-implantitis and peri-implant mucositis groups, which visited the dentist more regularly (Table 2).

### Participants' self-assessments

There was no statistically significant difference among the groups regarding the participants' most important reason for brushing their teeth, recognizing when they had problems with their implants, keeping appointments, or following dental recommendations (*p* > 0.05) (Table 3).

**Table 3:** Comparison of groups in terms of oral health evaluations

		Groups			Total	*p*
		Peri-implantitis	Peri-implant mucositis	Peri-implant health		
What is your most important purpose in brushing your teeth?	Preventing periodontal diseases and caries	12	12	6	30	0.649
		48.00%	60.00%	46.20%	51.70%	
	To make my teeth look cleaner by removing food residues from tooth surfaces	9	5	5	19	
		36.00%	25.00%	38.50%	32.80%	
	Prevent bad breath and have a fresher breath	0	1	0	1	
		0.00%	5.00%	0.00%	1.70%	
	To make my teeth look more beautiful	2	0	0	2	
		8.00%	0.00%	0.00%	3.40%	
	Not to lose my teeth	2	2	2	6	
		8.00%	10.00%	15.40%	10.30%	
How do you assess your current oral health?	Poor	6	4	3	13	0.028*
		24.00%	20.00%	23.10%	22.40%	
	Moderate	9	11	4	24	
		36.00%	55.00%	30.80%	41.40%	
	Good	9	3	6	18	
		36.00%	15.00%	46.20%	31.00%	
	Excellent	1	2	0	3	
		4.00%	10.00%	0.00%	5.20%	
Can you recognize yourself when you have a problem with the implant?	Yes	17	11	9	37	0.637
		68.00%	55.00%	69.20%	63.80%	
	No	8	9	4	21	
		32.00%	45.00%	30.80%	36.20%	
I suppose I've taken care of my mouth, including the implant	Yes	11	13	10	34	0.015*
		44.00%	65.00%	76.90%	58.60%	
	No	14	7	3	24	
		56.00%	35.00%	23.10%	41.40%	
Is it necessary to adhere to all scheduled appointments with your dentist for the whole duration of periodontal disease treatment?	Yes	25	18	12	55	0.447
		100.00%	90.00%	92.30%	94.80%	
	No	0	2	1	3	
		0.00%	10.00%	7.70%	5.10%	
Do you follow the dentist's recommendations?	Totally	19	13	8	40	0.563
		76.00%	65.00%	61.50%	69.00%	
	Partially	6	5	5	16	
		24.00%	25.00%	38.50%	27.60%	
	Never	0	2	0	2	
		0.00%	10.00%	0.00%	3.40%	
Which of the following is your reason for not caring for your mouth (including the implant) (brushing and cleaning the interdental)?	I do not think oral care is important	2	1	0	3	0.018*
		8.00%	5.00%	0.00%	5.20%	
	Individual inadequacies	0	3	0	3	
		0.00%	15.00%	0.00%	5.20%	
	It's hard to do it regularly	7	1	3	11	
		28.00%	5.00%	23.10%	19.00%	
	I forget	4	5	5	14	
		16.00%	25.00%	38.50%	24.10%	
	I will definitely do it	12	10	5	27	
		48.00%	50.00%	38.50%	46.50%	

*p>0.05

There was a statistically significant difference among the groups regarding the participants' evaluations of their current oral health status and their reasons for not performing oral care (*p* < 0.05). While the highest response rate was reasonable in the peri-implant health group, the highest response rate was moderate in the peri-implant mucositis group and moderate and reasonable in the peri-implantitis group. For the peri-implant health and peri-implant mucositis groups, "I forget" had the highest response rate. For the peri-implantitis group, "It is difficult to do it regularly" had the highest response rate (Table 3).

### Participants' knowledge and awareness levels

There was a statistically significant difference among the groups in terms of the answers given to the question "What is the factor that initiates implant-related diseases?" (*p* < 0.05). While the majority of the peri-implant health group responded that they did not know, the rates of responses of bacterial tooth attachment in the peri-implantitis group and malnutrition in the peri-implant mucositis group were high.

There was a statistically significant difference among the groups regarding the answer to the question "What is the most prominent symptom of implant-related diseases?'' (*p* < 0.05). Most of the individuals in the peri-implant health group responded with gingival bleeding, most of those in the peri-implantitis group responded with "I don't know", and most of those in the peri-implant mucositis group responded with gingival swelling.

There was no statistically significant difference among the groups regarding the answers to the questions of how many times per day and how many minutes should be spent per day (*p* > 0.05). In all the groups, the frequency was 2–3 times per day, and the duration was 1–2/2 min per session.

There was no statistically significant difference among the groups in terms of the answer given to the question "Is mouthwash used for bad breath?" (*p* > 0.05). The most frequent answer was "yes" in all groups.

There was no statistically significant difference among the groups regarding the answers to the question, "Are mouthwashes and antibiotics used for peri-implant diseases?'' (*p* > 0.05). The most frequent answer was yes in all groups.

There was no statistically significant difference among the groups in terms of the answer to the question "Does smoking affect the healing of periodontal diseases?" (*p* > 0.05). The most frequent answer was "yes" in all groups.

There was no statistically significant difference among the groups in terms of the answer to the question, "Do you know that systemic conditions that may adversely affect general body health, such as diabetes, heart disease, stroke, and pregnancy complications, can be a factor for periodontal and peri-implant diseases? (*p* > 0.05). The most frequent answer was "yes" in all groups.

There was a statistically significant difference among the groups regarding the answers to the question, "Do the medicines you use for general health affect oral hygiene and periodontal health?" (*p* < 0.05). The percentage of patients who answered yes was greater in the peri-implant mucositis group than the other grups.

There was a statistically significant difference among the groups regarding the answer to the question, "Is brushing alone sufficient for oral health?" (*p* < 0.05). In contrast to the other groups, the peri-implant mucositis group had a greater percentage of respondents who said "yes."

There was no statistically significant difference among the groups regarding the answer to the question "Does pregnancy affect periodontal disease?" (*p* > 0.05). The most frequent answer was "yes" in all groups.

There was no statistically significant difference among the groups in terms of the answer given to the question of whether all mouthwashes were used for more than two weeks (*p* > 0.05). In all groups, the most common answer was no.

There was no statistically significant difference among the groups regarding the answer to the question, "What is the tooth brushing technique used in the routine?" (*p* > 0.05). The most common response in all groups was, "I don't know."

There was no statistically significant difference among the groups regarding the answer to the question "How often should you go for routine dental check-ups?" (*p* > 0.05). The most common response in all groups was six months (Table 4).

**Table 4:** Comparison of groups in terms of general information of participants

		Groups			Total	*p*
		Peri-implantitis	Peri-implant mucositis	Peri-implant health		
What is the factor that initiates implant-related diseases?	Microbial dental plaque	7	4	2	13	0.014*
		28.00%	20.00%	15.40%	22.40%	
	Calculus	3	0	1	4	
		12.00%	0.00%	7.70%	6.90%	
	Malnutrition	1	6	0	7	
		4.00%	30.00%	0.00%	12.10%	
	Hereditary	1	3	1	5	
		4.00%	15.00%	7.70%	8.60%	
	Diabetes mellitus	2	2	1	5	
		8.00%	10.00%	7.70%	8.60%	
	I do not know	11	5	8	24	
		44.00%	25.00%	61.50%	41.40%	
What is the most prominent symptom of implant-related diseases?	Gingival bleeding	7	3	6	16	0,016*
		28.00%	15.00%	46.20%	27.60%	
	Swollen gingiva	3	6	2	11	
		12.00%	30.00%	15.40%	19.00%	
	Erythema	1	0	0	1	
		4.00%	0.00%	0.00%	1.70%	
	Oral malodor	3	2	1	6	
		12.00%	10.00%	7.70%	10.30%	
	I do not know	11	9	4	24	
		44.00%	45.00%	30.80%	41.40%	
How often should you clean your teeth in a day?	1 time	1	2	1	4	0.714
		4.00%	10.00%	7.70%	6.90%	
	2–3 times	21	15	10	46	
		84.00%	75.00%	76.90%	79.30%	
	3–5 times	0	2	1	3	
		0.00%	10.00%	7.70%	5.20%	
	I do not know	3	1	1	5	
		12.00%	5.00%	7.70%	8.60%	
How many minutes should you brush your teeth?	Less than 1 min	1	2	0	3	0.684
		4.00%	10.00%	0.00%	5.20%	
	1–2 min	9	7	3	19	
		36.00%	35.00%	23.10%	32.80%	
	2 min	4	5	4	13	
		16.00%	25.00%	30.80%	22.40%	
	2–3 min	4	4	4	12	
		16.00%	20.00%	30.80%	20.70%	
	More than 3 min	3	2	1	6	
		12.00%	10.00%	7.70%	10.30%	
	I do not know	4	0	1	5	
		16.00%	0.00%	7.70%	8.60%	
Is the hardness–softness of toothbrush bristles changed according to the periodontal problem?	Yes	22	17	13	52	0.405
		88.00%	85.00%	100.00%	89.70%	
	No	1	2	0	3	
		4.00%	10.00%	0.00%	5.20%	
	I do not know	2	1	0	3	
		8.00%	5.00%	0.00%	5.20%	
What is an effective way to prevent periodontal and peri-implant diseases?	Toothbrushing, dental floss and regular visits to the dentist	18	15	7	40	0.013*
		72.00%	75.00%	53.80%	69.00%	
	Proper nutrition	1	0	2	3	
		4.00%	0.00%	15.40%	5.20%	
	Only dental treatment	2	5	3	10	
		8.00%	25.00%	23.10%	17.20%	
	I do not know	4	0	1	5	
		16.00%	0.00%	7.70%	8.60%	
Is mouthwash employed for oral malodor?	Yes	19	14	9	42	0.378
		76.00%	70.00%	69.20%	72.40%	
	No	2	1	3	6	
		8.00%	5.00%	23.10%	10.30%	
	I do not know	4	5	1	10	
		16.00%	25.00%	7.70%	17.20%	
Are mouthwashes and antibiotics employed for peri-implant diseases?	Yes	18	13	9	40	0.991
		72.00%	65.00%	69.20%	69.00%	
	No	2	2	1	5	
		8.00%	10.00%	7.70%	8.60%	
	I do not know	5	5	3	13	
		20.00%	25.00%	23.10%	22.40%	
Does smoking affect the healing of periodontal diseases?	Yes	20	14	9	43	0.75
		80.00%	70.00%	69.20%	74.10%	
	No	1	3	2	6	
		4.00%	15.00%	15.40%	10.30%	
	I do not know	4	3	2	9	
		16.00%	15.00%	15.40%	15.50%	
Does clenching affect periodontal problems?	Yes	19	15	11	45	0.644
		76.00%	75.00%	84.60%	77.60%	
	No	2	3	0	5	
		8.00%	15.00%	0.00%	8.60%	
	I do not know	4	2	2	8	
		16.00%	10.00%	15.40%	13.80%	
Do you know that systemic conditions that may adversely affect general body health, such as diabetes, heart disease, stroke, and pregnancy complications, can be a factor for gingival and peri-implant diseases?	Yes	16	11	9	36	0.78
		64.00%	55.00%	69,20%	62,10%	
	No	4	6	2	12	
		16.00%	30.00%	15.40%	20.70%	
	I do not know	5	3	2	10	
		20.00%	15.00%	15.40%	17.20%	
Do the medicines you use for general health affect oral hygiene and periodontal health?	Yes	8	12	5	25	0.019*
		32.00%	60.00%	38.50%	43.10%	
	No	10	6	6	22	
		40.00%	30.00%	46.20%	37.90%	
	I do not know	7	2	2	11	
		28.00%	10.00%	15.40%	19.00%	
Can bacteria in the mouth be removed by brushing teeth?	Yes	19	14	8	41	0.621
		76.00%	70.00%	61.50%	70.70%	
	No	3	5	4	12	
		12.00%	25.00%	30.80%	20.70%	
	I do not know	3	1	1	5	
		12.00%	5.00%	7.70%	8.60%	
Is brushing alone sufficient for oral health?	Yes	3	7	3	13	0.021*
		12.00%	35.00%	23.10%	22.40%	
	No	20	10	7	37	
		80.00%	50.00%	53.80%	63.80%	
	I do not know	2	3	3	8	
		8.00%	15.00%	23.10%	13.80%	
Does pregnancy affect periodontal disease?	Yes	12	11	7	30	0.942
		48.00%	55.00%	53.80%	51.70%	
	No	4	3	1	8	
		16.00%	15.00%	7.70%	13.80%	
	I do not know	9	6	5	20	
		36.00%	30.00%	38.50%	34.50%	
Do birth control pills affect periodontal disease?	Yes	5	8	4	17	0.642
		20.00%	40.00%	30.80%	29.30%	
	No	5	4	3	12	
		20.00%	20.00%	23.10%	20.70%	
	I do not know	15	8	6	29	
		60.00%	40.00%	46.20%	50.00%	
Can all mouthwashes be used for more than two weeks?	Yes	3	3	1	7	0.946
		12.00%	15.00%	7.70%	12.10%	
	No	16	13	8	37	
		64.00%	65.00%	61.50%	63.80%	
	I do not know	6	4	4	14	
		24.00%	20.00%	30.80%	24.10%	
What is the tooth brushing technique used in the routine?	Roll Technique	2	0	1	3	0.435
		8.00%	0.00%	7.70%	5.20%	
	I do not know	23	20	12	55	
		92.00%	100.00%	92.30%	94.80%	
How often should you go for routine dental check-ups?	3 months	0	1	2	3	0.433
		0.00%	5.00%	15.40%	5.20%	
	6 months	15	11	5	31	
		60.00%	55.00%	38.50%	53.40%	
	6–12 months	7	5	2	14	
		28.00%	25.00%	15.40%	24.10%	
	1–2 years	0	1	1	2	
		0.00%	5.00%	7.70%	3.40%	
	2–6 years	3	2	3	8	
		12.00%	10.00%	23.10%	13.80%	

*p>0.05

## Discussion

The present study provides information about patients' oral hygiene habits, knowledge, and perceptions regarding dental implants in a sample of patients previously treated with dental implants.

We discovered that the frequency of tooth brushing among children positively correlated with maternal work status and the number of children in the household, even after we adjusted for confounding variables. Furthermore, there was a strong correlation between the paternal educational attainment and the age at which teeth brushing began [34]. The oral hygiene status of their children was unknown to many parents. Consequently, it is important to offer educational treatments to young families to improve their ability to identify their child's dental needs [35]. On the other hand, Ashoori et al. (2021) reported that maternal education did not have any additional effects on the students' education [36]. The educational status of the patients' mothers was equivalent across the three groups.

Using a toothbrush is the most critical measure for ensuring oral hygiene [37]. Manual tooth brushing was the most commonly used oral hygiene procedure, with 77.6% [22]. Approximately 40.5% of patients adhered to a regular toothbrush change schedule every three months [38]. A total of 33.8% of periodontitis patients position the brush at the tooth-gingiva junction, first move it back and forth, and then perform a sweeping movement towards the coronal [39]. Specifically, 66% of the implants were cleaned using a powered toothbrush, whereas 44% were cleaned via a manual toothbrush [17]. In this study, toothbrushes were used by 35.5% of the patients with implants, while powered toothbrushes were used by 3.9%; powered toothbrush use was at the highest level in patients with peri-implant health. The patients changed their toothbrushes at most every three months 37.10%. 43.10% of the patients brushed from the tooth-gingiva junction to the crown.

The presence of plaque/calculus and a lack of interproximal cleaning were found to be strongly linked to peri-implant illness [40]. The peri-implant status correlates with the accessibility of self-performed proximal hygiene [17]. The majority of patients claimed that they used nothing at all for their implants. The percentage of people who utilized interdental brushes for their implants was 9.2%, and the distribution was consistent across every group.

Tooth brushing partially reduces plaque formation in the interdental region while flossing increases plaque removal [37]. While flossing was not used as an element of oral hygiene for 46% of the implants assessed, the use of interdental brushes was limited to 48% of the implants, and in most cases, the time spent using them was 1 min. The oral hygiene device least employed around implants was the water flosser, which was used for only 4.7% of the implants, and in the vast majority of cases (56.6%), oral mouthwashes were not used as a complementary oral hygiene procedure [17]. Regarding daily interproximal cleaning, 27% of the patients performed it [41]. Brunello et al. (2020) reported the frequency of interdental brush usage to be 64.2%, whereas the frequency of utilizing dental floss was 59.4% [22]. Dental floss was employed by 11.2% of the individuals in this study.

Toothpaste is a material, either in the form of a paste or gel, applied to a toothbrush to preserve and enhance oral health and aesthetics [42]. Almost all participants (97.9%) indicated that toothpaste was the most commonly used product for oral hygiene [22]. Eighty-two percent of the individuals with periodontal disease used fluoride toothpaste [39]. The study revealed a toothpaste utilization rate of 35%.

Professional guidelines for individual oral hygiene often involve brushing one's teeth using a mild amount of pressure at least per day for 2–3 min [43]. In most cases, patients reported a brushing frequency of three times per day (2 min each session) with a powered toothbrush [17]. In another study, 86% of participants brushed their teeth twice daily [41]. Buunk-Werkhoven et al. (2011) reported that 82.8% of patients brushed their teeth twice per day or more than twice per day [32]. The percentage of patients who brushed their teeth for two minutes or more was 31%. The lowest percentage of patients who brushed their teeth for more than two minutes was found in the group with peri-implantitis.

Regular visits for implant maintenance included assessing the level of oral hygiene and scaling the implant if necessary [44]. Olerud et al. (2012) reported that 92% of the participants received routine dental care [41]. A total of 40.8% of the participants reported visiting a dentist. 21.9% of the participants visited their doctor once per year, while 67.6% went to doctor appointments every six months or less [22]. In this study, one visit per year according for 32.9% of the patients. Patients with peri-implantitis and peri-implant mucositis visited the dentist more often.

Peri-implant disorders are typically symptomatic and are undetected by patients [45]. A total of 25.5% of the patients affirmed that they visited a dentist only if there was any complication [22]. 47% of dental students go to the dentist when they have a toothache instead of going for regular check-ups [46]. A total of 56.9% of the patients presented with toothaches. In the group with peri-implantitis, 72% of the patients presented with toothache.

Professional oral hygiene instruction is vital for optimal treatment effectiveness [47]. More than 90% of the participants declared that they had received instruction about oral hygiene regarding implants, and only 40.4% reported having tried oral hygiene tools at the office [22]. Oral hygiene education was most common in patients with peri-implant health at 76.9%.

We now know that peri-implant disease points to a condition that, although sharing some characteristics with periodontal disease, is likely far more complicated and has specific characteristics that need more study [48]. A total of 44.6% of the participants believed that they had periodontal disease [49]. Peri-implant diseases are, in most cases, asymptomatic and not perceived by the patients [45]. Participants mainly evaluated their situation as moderate. A total of 46.20% of peri-implant health patients stated that the condition of their implants was good.

Oral health knowledge is considered to be an essential prerequisite for health-related practices and better oral health [20]. In the case of plaque-associated peri-implantitis, local contributors, including surgical and prosthetic factors and soft and hard tissue characteristics, may be factors predisposing individuals to biofilm adherence around dental implants, thus leading to inflammation [50]. A total of 26.8% of the participants believed that plaque is a factor affecting periodontal disease [51]. In this study, 22.40% of individuals said that diseases around the implant were related to plaque. Patients with peri-implantitis gave the highest percentage of "yes" answers.

Good oral hygiene must be performed to maintain peri-implant health [52]. Increased overall plaque scores significantly correlated with peri-implant disease, whereas a very inadequate oral hygiene condition had a strong association with peri-implantitis [53]. According to the survey, 69% of the respondents reported utilizing toothbrushes, dental floss or interdental brushes, and frequent dental appointments as preventive measures against dental and implant-related disorders. The group diagnosed with peri-implantitis had the largest proportion of patients providing "yes" responses.

Higher levels of smoking intensity have been associated with a higher risk of developing peri-implantitis [54]. A total of 20% of the participants said that smoking would affect the implant [55]. The percentage of university students who think that smoking affects periodontal disease varies from 60 to 80%, depending on the field of study [33]. A total of 74.10% of the participants thought that smoking would affect the area around the implant.

Recognizing the significance of biofilms in individuals with peri-implant diseases and considering systemic variables are crucial for effective treatment planning and maintaining peri-implant health. Current evidence indicates an association between inflammatory peri-implant diseases and systemic inflammation [56]. Ken et al. (2017) found that 19% of participants thought there was a relationship between systemic diseases and implants [55]. More than half of university patients think that diabetes affects periodontal status, while heart disease does not [33]. In this study, 62.10% of the participants thought that systemic diseases affect the implant environment. Individuals with peri-implant health, accounting for 69.20% of the sample, believed that systemic diseases could affect the peri-implant area.

Bleeding on probing in the peri-implant area may indicate inflammation and is a health indicator when no bleeding is observed [57]. Approximately 66% of individuals believed that bleeding gums of inflammation [58]. In this study, 27.60% of the participants said that bleeding was a symptom of disease around the implant, 46.20% of the individuals with peri-implant health responded "yes" to this question.

The absence of regular maintenance treatment is a risk factor/indicator for peri-implantitis [7]. Recall maintenance visits should be arranged every three months for the first year following treatment, after which they should be modified to meet the patient's needs. Individuals with poor oral hygiene, heavy deposits, and disease susceptibility need more regular follow-up care, whereas individuals with good oral hygiene, few deposits, and disease resistance need less frequent professional hygiene maintenance [59] . In patients with a high standard of oral hygiene and who were enrolled in a recall system every six months, the peri-implant conditions obtained following peri-implant surgery were stable for most patients and implants for five years [60]. No significant difference was found among the groups regarding how often an individual should visit the dentist for implant control. The most common answer in all groups was to visit every six months.

## Conclusions

Although the use of gum protection toothpaste was significantly higher in the peri-implant group, oral hygiene habits and the use of products were not significantly different in patients with implants. Although they were knowledgeable about some issues related to peri-implant disease in the peri-implant health group, similar answers were given in all groups, and awareness was high. However, this awareness needed to be reflected in behaviors. Therefore, as clinicians, we need to take a more active role in developing oral hygiene habits in patients with implants by giving more oral hygiene control appointments.

## Data Availability

The datasets used and/or analysed during the current study available from the corresponding author on reasonable request.

## Abbreviations

ANOVA: One-way analysis of variance
STROBE: The strengthening the reporting of observational studies in epidemiology
UNC: University of North Caroline
SPSS: Statistical package for the social sciences
BMI: Body mass index value
